# Changing professional behaviours: mixed methods study utilising psychological theories to evaluate an educational programme for UK medical doctors

**DOI:** 10.1186/s12909-021-02510-4

**Published:** 2021-02-05

**Authors:** Asta Medisauskaite, Ann Griffin, Rowena Viney, Ahmed Rashid, Antonia Rich

**Affiliations:** 1 Research Department of Medical Education, UCL Medical School, Royal Free Hospital, Room GF/664, London, NW3 2PF UK; 2 Research Department of Medical Education, UCL Medical School, 74 Huntley Street, London, WC1E 6AU UK

**Keywords:** Professionalism, Intervention, Theory of planned behaviour, Raising concerns, Reflection

## Abstract

**Background:**

The Theory of Planned Behaviour (TPB) has been proposed as a useful framework to investigate professional behaviour, however, was not yet applied to the evaluation of an educational intervention. This study will address this gap by utilising the TPB to evaluate the effectiveness of an education programme delivered by the professional regulator for UK doctors in enhancing three professional behaviours: raising concerns, engaging in reflective practice, and use of regulator confidentiality guidance.

**Methods:**

This is a comprehensive mixed methods study combining qualitative (interviews) and quantitative (quasi-experiment) data. Intervention participants were asked to complete a survey measuring the variables in the TPB (attitudes, subjective norms, perceived behavioural control, and intention) for the three professional behaviours before, immediately post, and 3-months later following the education programme. Ninety-four doctors completed the survey pre/post intervention and 38 at all three times. One hundred and eleven doctors from the same hospital trust who did not take part in the intervention completed the survey at two time points and formed the control group. Forty-two interviews were conducted with intervention participants.

**Results:**

The quantitative study revealed that the educational intervention significantly improved attitudes (raising concerns, using confidentiality guidance), subjective norms (raising concerns, reflective practice, using confidentiality guidance), perceived control (raising concerns, using confidentiality guidance), and intentions (using confidentiality guidance) (Group and Time interaction; *Fs* ≥ 3.996, *ps* ≤ .047, ηp^2^ ≥ .020). Non-UK graduate doctors’ subjective norms towards raising concerns and confidentiality guidance increased significantly after the intervention (*Fs* ≤ 6.602, *ps* ≥ .011, ηp^2^ = .032 *F* = 6.602, *p* = .011, ηp^2^ = .032), but not UK graduates (*p* > .05). Interviews revealed that doctors had positive views about professional behaviours but also mentioned numerous barriers to actually engage in more complex, context dependent behaviours.

**Conclusions:**

This study demonstrates that an educational intervention was successful in improving the TPB variables of three professional behaviours. It also revealed that teaching professionalism does not happen in isolation and, therefore, personal and contextual factors are crucial to consider. To change complex professional behaviours, barriers at all levels i.e., personal, organisational and system, should be addressed.

**Supplementary Information:**

The online version contains supplementary material available at 10.1186/s12909-021-02510-4.

## Background

The topic of professionalism has been of great interest in medical education for a couple of decades now. Despite that, a systematic review seeking to collect evidence for how professional behaviour should be taught in medical education found no unifying theoretical or practical model that could be used for integrating the teaching of professionalism into medical education [1]. The socio-psychological theory of planned behaviour (TPB) [2] has been proposed as a useful framework to help evaluate unprofessional behaviour [3]. The TPB has been widely used in the last three decades and meta-analyses have shown it can predict a number of health-related behaviours [4–7]. The TPB has also been applied in educational settings, including laboratory learning [8], intention to pursue higher education [9], and teachers’ intentions to implement national reform standards [10]. In light of the success of using the TPB in the health and education fields, this study evaluates the effectiveness of an education programme for UK medical doctors in enhancing their professional behaviours using TPB as the theoretical background for evaluation.

The TPB posits that intentions are the precursor of behaviours, and that three psychological factors predict intentions: 1) attitudes, 2) subjective norms, and 3) perceived behavioural control. Attitudes are a person’s overall evaluation of the behaviour and include their beliefs about the outcome and consequences; subjective norms are a person’s estimation of the social pressure to perform or not perform the behaviour; perceived behavioural control is the extent to which a person feels able to perform the behaviour, and is determined by control beliefs about the power of external and internal factors that inhibit or facilitate the behaviour [4]. Reliable predictions about the likelihood of a person enacting a particular behaviour can therefore be made on the basis of knowledge about that person’s attitudes, subjective norms, perceived behavioural control, and behavioural intentions in relation to the behaviour [2]. This theory was previously used in analysing professionalism and showed predictive value of three professional behaviours: intention to engage in raising concerns, reflective practice, and use of confidentiality guidance [11].

The literature reveals a diverse and contested range of definitions and conceptualisations of professionalism [12]. There is no overall agreement about what professionalism is beyond being a varied integrated complex of attitudinal and behavioural characteristics [13]. Whilst there are a few studies measuring professional behaviours [14, 15], there are no studies applying the TPB to evaluate the effectiveness of a professional educational intervention for doctors. This study will address this gap by utilising the TPB to evaluate a professional education programme’s impact on three professional behaviours: raising a patient safety concern, engaging in reflective practice, and use of regulator confidentiality guidance. As part of a General Medical Council (GMC) funded programme, this is a comprehensive mixed methods study combining qualitative examination of doctors’ evaluation of professional behaviours and quantitative investigation of the effectiveness of an intervention. This is a unique research studying an intervention in the real world context and exploring the factors that influence change. This paper will briefly summarise findings from the qualitative research focusing on the evaluation of professional behaviours to give the reader a holistic picture of the factors that influence doctors’ professional behaviours (for further information the qualitative study is discussed in other publications) [16, 17]. The main focus of this paper is the design and results of the quantitative arm, drawing on the qualitative research where relevant to aide interpretation of the quantitative results.

## Methods

The GMC’s Duties of a Doctor (DoaD) was a preventative education programme ran at hospitals across the UK. The programme typically consisted of five or six half-day workshops ran over the course of 4 to 6 months and facilitated by Regional Liaison Advisors (RLAs; members of the GMC’s Regional Liaison Service, RLS) who were from various backgrounds (exclusively non-medical). The workshops aimed to influence doctors’ professional behaviours by providing information on guidance on the regulator’s standards, and discussing how they should be applied, particularly in challenging and ambiguous situations (more information about the workshop - [18]).

This study is a mixed methods study consisting of qualitative (interviews with DoaD participants) and quantitative (pre/post design survey with comparator group) data. The project was approved by the UCL Research Ethics Committee, ref. 5490/001.

### Qualitative part

#### Procedure

Data was collected between March and August 2017. The study purposefully sampled from seven RLS regions across England, and from DoaD programmes at different NHS Trust sites which were delivered to various cohorts of doctors (from a variety of career stages, place of primary medical qualification, primary and secondary care). This maximum variation sample aimed to capture the broadest range of views and enabled an examination of the contextual and individual influences affecting the DoaD programme, as well as an exploration of the consistency and any variability of the intervention.

All focus groups and most interviews were conducted immediately after a session, with some telephone interviews arranged at a later date (interview guide is presented elsewhere) [17]. Sampling ensured a spread in terms of the doctors’ characteristics for the four different topics discussed: evaluation of the DoaD workshops, use of GMC confidentiality guidance, raising concerns, and reflective practice. The focus groups and interviews followed a semi-structured guide and were audio recorded and transcribed professionally.

#### Analysis

The interviews were independently coded by three team members (AG, AR, RV) using QSR NVivo 11 who met regularly to ensure consistency. Interview transcripts were subjected to thematic analysis [19]. An analytic framework was developed using the TPB for overarching deductive themes, however researchers also approached the data inductively to allow any further themes and sub-themes to emerge that were not captured by the TPB. Data underwent analysis using an initial coding scheme which was developed based on analysing two transcripts. Each of the team members coded the same transcripts, and the comparison and discussion about these was used to devise the first iteration of the coding framework. Thereafter, the remaining transcripts were distributed between the team members for coding.

### Quantitative methods

This part of the study was a quasi-experimental study using a pre/post (Time-1/Time-2) questionnaire and 3-month follow-up (Time-3; intervention group only). Data were collected via paper and online between September 2017 and February 2019, in 12 RLS Regions in England.

#### Procedure

Doctors attending the DoaD programmes were invited to take part in the study prior to the first session by the RLAs facilitating the course (paper questionnaire) or email (containing a link to Online survey platform). Participants were asked to complete the questionnaire again at the end of their last session (Time-2) and 3 months later (Time-3). Doctors who were from the same Trust (a hospital or group of hospitals in the National Health Service serving a geographic area) as intervention group participants but who did not take part in the DoaD programme were emailed an invitation to take part in this study online (Online Survey platform). The control group of doctors completed the questionnaire on the same time as DoaD participants (Time-1). Control participants were asked to complete the questionnaire 3 months after the end of the DoaD programme at that Trust (Time-2). We asked participants for the identifiable information to be able to link Time-1 and follow-up questionnaires together; this information was separated from the rest of the questionnaire and each questionnaire was given a code (paper – identifiable information was provided on the first perforated page making it easy to separate this information; online – spreadsheets were anonymised). Participants were informed about the use of identifiable information prior taking part. The process was fully compliant with General Data Protection Regulation (2018).

#### Questionnaire design

The questionnaire, designed bespoke for the programme was based on guidance for constructing a TPB questionnaire [20], measured three professional behaviours (raising concerns, reflective practice, and using confidentiality guidance) in the four TPB dimensions (full description in Table 1 and [11]; the questionnaire is presented in Supplementary file 1):

1. **Attitudes.** The doctor’s overall evaluation of the behaviour.
2. **Subjective norms**. The degree of pressure felt from various organisations and people to act in a certain way (e.g., peers).
3. **Perceived behaviour control**. Doctors’ confidence and beliefs about their ability to carry out the behaviour.
4. **Intentions**. The extent to which doctors’ intend to carry out the behaviour in the future.

**Table 1:** Questionnaire Measures

Measures	Scale	Cronbach α	No of items	Example item
Use of confidentiality guidance	Attitude	0.842	8	Overall, I think that the GMC confidentiality guidance is hard to apply—easy to apply
	Subjective norm	0.944	11	It is expected of me to use the GMC confidentiality guidance
	Perceived behavioural control	0.739	4	I have enough time to refer to the GMC confidentiality guidance
	Intention	0.856	3	I intend to refer to the GMC confidentiality guidance the next time I’m uncertain
Raising Concerns	Attitude	0.659	4	Overall, I think raising a concern is worthless – worthwhile
	Subjective norm	0.838	11	It is expected of me that I report a concern if I have one
	Perceived behavioural control	0.634	2	I am confident that I can raise a concern if I want to
	Intention	0.779	3	I want to raise a concern when I have one in my work environment
Reflection	Attitude	0.883	8	Reflecting on my practice makes me a better doctor
	Subjective norm	0.875	12	People who are important to me think I should reflect on my practice
	Perceived behavioural control	n/a^a^	1	For me to reflect on my practice is difficult - easy
	Intention	0.823	3	I intend to reflect on my practice

Attitudes, subjective norms, perceived behaviour control, and intentions were measured on a 7-point bipolar or Likert scale scored from 1 to 7 (as suggested in the guide for constructing a TPB questionnaire [20]). Higher scores showed more positive attitudes, norms, perceived control, and intentions. A couple of items were excluded to improve scales internal consistency (initial Cronbach’s α = −.05 to α = .46, see more details in [11]).

The questionnaire also measured demographic characteristics (location of work, gender, ethnicity, grade, and years of experience working as a doctor in the UK).

#### Analysis

Statistical analyses were performed with SPSS v24. For each participant the mean scores of non-missing items were imputed to replace missing items if there were less than a third of missing items forming the scale. When more than a third of items were missing the individual score for the scale was deleted. Independent sample Student’s t-test was used to compare mean scores of all scales at baseline between intervention and control groups. Doctors attending the DoaD had significantly more positive attitudes towards raising concerns and stronger intentions to use confidentiality guidance (approachability and role; see Supplementary file 2: Table 2; *t* ≥ − 2.630, *p* ≤ .009).

**Table 2:** Summary of qualitative results

**Use of confidentiality guidance**	
**Summary**: Participants had largely positive attitudes towards the GMC’s confidentiality guidance - as a support for them in their work (attitudes). ‘Everybody approved’ of confidentiality guidance (subjective norms). The barriers to using the guidelines were primarily to do with the length of the guidance, and a lack of practical support in maintaining confidentiality in the workplace (perceived behavioural control). Nevertheless, attending the DoaD programme increased knowledge of the confidentiality guidance and confidence in applying it. Some doctors said that as a result of the DoaD programme they would now be more likely to intend to refer to the confidentiality guidelines for difficult cases, such as whether or not do disclose information to the police (intentions).	
**Indicative quotes:**	
Attitudes:	*It’s the standard advice from the regulator, so if you follow it, it can’t be wrong. And it’s good to have that framework. I think it is so much more clearly written now than it was. -* ***GP-Consultants****/****UK graduate***
Intentions	*Yes, I don’t think it’s going to change my practice but I think it’s given me more confidence that I was doing the right thing in the first place and to carry on making the same decisions. –* ***Foundation Year 2 Doctors/UK graduate***
**Raising a concern**	
**Summary:** Raising a concern was considered an appropriate professional attitude particularly in the face of patient safety issues; however, participants expressed unease about *actually* raising a concern. Having attended DoaD, participants felt that the RLA, and thus the GMC, understood the challenges around the reality of raising a concern (attitudes). Some felt that colleagues would give ‘lip service’ to the need to raise a concern, verbalising approval in principal, but retreating when it became a reality (subjective norms). Some doctors had raised concerns, but due to lack of action and/or feedback, had ceased to raise them. Barriers to raising a concern were substantial and operated at the level of the individual, interpersonal (e.g., potential to cause dysfunctional relationships between colleagues) and organisational (e.g. organisational culture, a lack of supportive leadership) (perceived behavioural control). Foundation doctors expressed that they would prefer to raise an issue with a senior as a first step instead of referring to the GMC guidance. More senior doctors expressed that having all attended the same course, they had gained greater empowerment to raise a concern within their organisation as they could garner support from one another (intentions).	
**Indicative quotes:**	
Attitudes	*So morally, you might know exactly what you want to do, ethically, you know what you should be doing, but you don’t have the role models of ‘do this because it’s for the common good, and professionally it is a good thing to do’. That doesn’t happen. -* ***Consultant-SAS doctors/UK graduate***
Subjective norms	*They [management] don’t enable, I don’t think that they enable. I don’t think there are barriers put in place, but there’s not a culture … we are not enabled to raise concerns.* ***- Consultant-SAS doctors/UK graduate***
**Reflective practice**	
**Summary:** Reflection was mostly seen as a positive activity and of benefit to doctors and patients alike, but there were some criticisms of how it is expected to be done. Negative attitudes were about formal reflection which was reported as mandatory and a ‘tick box exercise’ (attitudes). Reflection was felt to be universally positive by others (subjective norms). There were several barriers to carrying out reflection: time, high demands of service delivery, lack of feedback on the quality of written reflection, lack of training, and the absence of a positive workplace culture for disclosing mistakes (perceived behavioural control). Participants generally did not feel that they would reflect more or differently now that they had attended the DoaD programme as they already felt they reflected sufficiently. However the course reinforced the benefits of reflection and as such served to encourage participants to continue to reflect (intentions).	
**Indicative quotes:**	
Perceived behavioural control	*The other thing is that sometimes when you reflect more in depth, when you have time, you haven’t got anywhere else to go then to discuss your reflections. So, of course, you can look up things yourself, you know, but it’s like you’re just left on your own; you, because of the how the system is, so as an SAS doctor, you haven’t got any time to discuss with colleagues or … and that leaves it a bit unfinished sometimes. –* ***SAS doctor/non-UK graduate***
Intentions	*I don’t think so, it’s just something else to reflect on, I think we all do reflect so I don’t think it will make any difference particularly to how I approach things. But it will – I think I will reflect on it, if that makes any sense, I don’t think it will be life changing in terms of that. I guess because I’ve already done quite a lot of work on it, if that makes sense.* ***- GP/UK graduate***

*Note. SAS* Specialty and Associate Specialist

Sociodemographic characteristics were compared between groups (control and intervention) at baseline using the Chi Square test. Doctors’ demographic characteristics (ethnicity, primary medical qualification (PMQ), role and work experience) significantly differed between intervention and control groups (see Table 3; χ^2^ ≥ 21.549; *p* < .001) and, therefore, we adjusted for PMQ (as it correlated with ethnicity but had a lower number of categories) and years of experience (as it correlated with role but was a scale and not a nominal variable) in subsequent analysis.

**Table 3:** Demographic characteristics of study participants

		***n*** (%)			Statistics
	LRMP	Total	Control	DoaD	
Gender					
Male	54.5%	92 (44.9%)	48 (51.1%)	44 (40.0%)	χ2(2) = 3.916; *p* = .141
Female	45.5%	111 (54.1%)	45 (47.9%)	66 (60.0%)	
Prefer not to say		1 (0.5%)	1 (1.1%)	-	
Ethnicity					
White	52.4%	141 (68.8%)	80 (85.1%)	61 (55.0%)	χ2(2) = 21.549; *p* < .001
BME	31.8%	55 (26.8%)	12 (12.8%)	43 (38.7%)	
Prefer not to say/other	15.8%	9 (4.4%)	2 (2.1%)	7 (6.3%)	
Region of primary medical qualification					
UK	63%	132 (64.4%)	80 (85.1%)	52 (46.8%)	χ2(1) = 32.493; *p* < .001
Non-UK	37%	73 (35.6%)	14 (14.9%)	59 (53.2%)	
Role					
Consultant	31.6%	100 (48.8%)	67 (71.3%)	33 (29.7%)	χ2(2) = 42.904; *p* < .001
Trainees	21%	36 (17.6%)	16 (17.0%)	20 (18.0%)	
Other (e.g, Staff Grade, Associate Specialist, etc.)	23.3%	68 (33.7%)	11 (11.7%)	58 (52.3%)	
Experience working as a doctor in the UK (years)					
< 1	n/a	35 (17.1%)	3 (3.2%)	32 (28.8%)	χ2(4) = 44.010; *p* < .001
1–4	n/a	29 (14.1%)	8 (8.5%)	21 (18.9%)	
5–10	n/a	22 (10.7%)	9 (9.6%)	13 (11.7%)	
11–20	n/a	57 (27.8%)	28 (29.8%)	29 (26.1%)	
> 21	n/a	62 (30.2%)	46 (48.9%)	16 (14.4%)	
**Total number**		**205**	**94**	**111**	

*Note*. *LRMP* List of Registered Medical Practitioners, *BME* Black and minority ethnic background

Split-plot (mixed-design) ANCOVA (with Bonferroni correction accounting for multiple testing) tested the effectiveness of the intervention (within/between subjects). The results section will focus on this analysis. Repeated measures ANOVA (with Bonferroni correction) was used to measure the change between three time points for doctors who received the intervention. Sphericity was assumed (Mauchly’s Test *p* > .05) for all scales. The full results of repeated measures are presented in Supplementary material 5.

## Results

### Qualitative analysis

Forty-two DoaD programme participants across all seven sites took part in one-to-one interviews or focus groups (Supplementary file 3). The qualitative framework, developed from the TPB, is common to the three behaviours (use of confidentiality guidance, raising a concern, and reflection) and takes the following overarching themes from the TPB: attitudes, subjective norms, perceived behavioural control, and behavioural intentions. A brief summary of the qualitative findings is presented below (Table 2). For further details please see the paper on raising concerns [16] and the report [17].

### Quantitative analysis

Ninety four doctors attending the DoaD programme (from 223 at Time-1; 42%) and 111 doctors in the control group (from 239 at Time-1; 46%) took part in the study at two time points (see flow diagram presented in Supplementary file 4). The total of 54% of participants were female, 69% white, 49% consultants, and 64% UK graduates (Table 3). Demographic characteristics of doctors in this study were similar to doctors on the List of Registered Medical Practitioners (containing details of all registered doctors in the UK), except for ethnicity and grade: higher number of white ethnicity doctors and consultants participated in this study. A summary of the results can be found in Table 4 (see Supplementary file 5: Table 2 for the analysis of 3 months follow up).

**Table 4:** The main results from the split-plot ANCOVA

	Control (***M***)			Intervention (***M***)			Interaction effect of Group X Time	Interaction effect of Group x Time x PMQ
	Time-1	Time-2	Marginal Mean scores	Time-1	Time-2	Marginal Mean scores		
**Confidentiality guidance**								
Attitudes	4.59	4.65	0.053 (*F* = 0.146, *p* = .703, ηp^2^ = .001)	4.53	4.99	**0.466 (*F*** **= 23.513,** ***p*** **< .001, ηp**^**2**^ **= .107)**	***F*(1,197) = 5.596,** ***p*** **= .019, ηp**^**2**^ **= .028**	*F*(1,197) = 3.067, *p* = .081, ηp^2^ = .015
Subjective norms	4.28	4.03	−0.247 (*F* = 1.476, *p* = .226, ηp^2^ = .007)	4.30	4.68	**0.381 (*F*** **= 7.210,** ***p*** **= .008, ηp**^**2**^ **= .035)**	***F*(1,196) = 5.599,** ***p*** **= .015, ηp**^**2**^ **= .030**	***F*(1,196) = 4.937,** ***p*** **= .027, ηp**^**2**^ **= .025**
Perceived behaviour control	4.40	4.17	−0.228 (*F* = 2.670, *p* = .104, ηp^2^ = .013)	4.50	5.04	**0.545 (*F*** **= 31.569,** ***p*** **< .001, ηp**^**2**^ **= .139)**	***F*(1,196) = 19.396,** ***p*** **< .001, ηp**^**2**^ **= .090**	*F*(1,196) = 0.412, *p* = .522, ηp^2^ = .002
Intentions^a^	5.10	4.74	**−0.362 (*F*** **= 4.009,** ***p*** **= .047, ηp**^**2**^ **= .020)**	5.46	5.74	**0.274 (*F*** **= 4.797,** ***p*** **= .030, ηp**^**2**^ **= .024)**	***F*(1,197) = 7.827,** ***p*** **= .006, ηp**^**2**^ **= .038**	*F*(1,197) = 0.028, *p* = .867, ηp^2^ < .001
**Raising concerns**								
Attitudes	3.97	3.89	−0.079 (*F* = 0.280, *p* = .597, ηp^2^ = .001)	4.24	4.58	**0.338 (*F*** **= 10.905,** ***p*** **= .001, ηp**^**2**^ **= .052)**	***F*(1,198) = 4.991,** ***p*** **= .027, ηp**^**2**^ **= .025**	*F*(1,198) = 1.153, *p* = .284, ηp^2^ = .006
Subjective norms	4.89	4.71	−0.174 (*F* = 1.506, *p* = .221, ηp^2^ = .008)	4.66	4.89	**0.232 (*F*** **= 5.696,** ***p*** **= .018, ηp**^**2**^ **= .028)**	***F*(1,198) = 5.238,** ***p*** **= .023, ηp**^**2**^ **= .026**	***F*(1,198) = 8.510,** ***p*** **= .004, ηp**^**2**^ **= .041**
Perceived behaviour control	5.35	5.23	−0.115 (*F* = 0.391, *p* = .532, ηp^2^ = .002)	5.15	5.50	**0.347 (*F*** **= 7.475,** ***p*** **= .007, ηp**^**2**^ **= .036)**	***F*(1,198) = 3.996,** ***p*** **= .047, ηp**^**2**^ **= .020**	*F*(1,198) = 0.005, *p* = .946, ηp^2^ < .001
Intentions^a^	5.72	5.59	−0.139 (*F* = 0.696, *p* = .405, ηp^2^ = .004)	5.51	5.75	**0.234 (*F*** **= 4.170,** ***p*** **= .042, ηp**^**2**^ **= .021)**	*F*(1,198) = 3.190, *p* = .076, ηp^2^ = .016	*F*(1,198) = 0.464, *p* = .497, ηp^2^ = .002
**Reflection**								
Attitudes^a^	5.25	5.08	−0.270 (*F* = 3.864, *p* = .051, ηp^2^ = .019)	5.35	5.47	0.115 (*F* = 1.485, *p* = .224, ηp^2^ = .007)	***F*(1,198) = 5.005,** ***p*** **= .026, ηp**^**2**^ **= .025**	*F*(1,198) = 0.022, *p* = .882, ηp^2^ < .001
Subjective norms	4.45	4.37	−0.073 (*F* = 0.264, *p* = .608, ηp^2^ = .001)	4.67	5.05	**0.378 (*F*** **= 14.953,** ***p*** **< .001, ηp**^**2**^ **= .070)**	***F*(1,198) = 6.410,** ***p*** **= .012, ηp**^**2**^ **= .031**	*F*(1,198) = 1.351, *p* = .247, ηp^2^ = .007
Perceived behaviour control	5.06	5.00	−0.056 (*F* = 0.057, *p* = .811, ηp^2^ < .001)	5.01	5.08	0.069 (*F* = 0.182, *p* = .671, ηp^2^ = .001)	*F*(1,196) = 0.181, *p* = .671, ηp^2^ = .001	*F*(1,196) = 0.106, *p* = .746, ηp^2^ = .001
Intentions^a^	6.02	5.71	**−0.313 (*F*** **= 4.122,** ***p*** **= .044, ηp**^**2**^ **= .020)**	6.10	6.28	0.182 (*F* = 2.940, *p* = .088, ηp^2^ = .015)	***F*(1,198) = 6.557,** ***p*** **= .011, ηp**^**2**^ **= .032**	*F*(1,198) = 0.980, *p* = .323, ηp^2^ = .005

*Note*. ^a^ Additional analysis is presented in Supplementary material 5; *PMQ* Primary medical qualification

### Three professional behaviours and TPB factors

Doctors attending the DoaD programme had a significant increase in attitudes (raising concerns, using confidentiality guidance), subjective norms (raising concerns, reflective practice, using confidentiality guidance), perceived control (raising concerns, using confidentiality guidance), and intentions (using confidentiality guidance) (significant Group and Time interaction; *Fs* ≥ 3.996, *ps* ≤ .047, ηp^2^ ≥ .020).

Even though there were significant Time and Group interactions, the increase in attitudes and intentions towards reflection in the intervention group was not significant (*p* > .05), but the decrease in these measures was significant (or borderline significant *p* = .051) in the control group, which might have resulted in the significant main effect (see Supplementary file 6 for additional analyses of the change scores for these measures).

In addition, intentions to raise concerns significantly increased after the programme (*F* = 4.170, *p* = .042, ηp^2^ = .021), but this change was not significantly different from the change in the control group (*F* (1,198) = 3.190, *p* = .076, ηp^2^ = .016). To investigate further, additional analysis (Supplement material 6) of the change scores (Time-2 - Time-1) was performed and showed no significant difference between groups (*p* > .05).

The positive change after the intervention in doctors’ attitudes towards the use of confidentiality guidance and subjective norms towards reflective practice persisted over time (e.g. 3 months, *p* < .05; see Supplementary file 5).

### Effect of PMQ

There was a significant Group, Time and PMQ interaction when analysing subjective norms towards raising concerns and using confidentiality guidance (*Fs* ≤ 8.510, *ps* ≥ .004, ηp^2^ ≤ .041, see Table 4). Among non-UK graduates, doctors’ subjective norms towards raising concerns and confidentiality guidance increased significantly in the intervention group (*Fs* ≤ 6.602, *ps* ≥ .011, ηp^2^ = .032 *F* = 6.602, *p* = .011, ηp^2^ = .032), but not in UK graduates (*p* > .05) (see Supplementary file 5).

## Discussion

Teaching professionalism is an important part of medical education. This study investigates if an educational intervention (the Duties of the Doctor programme, DoaD) is effective in improving three professional behaviours: use of the regulator’s confidentiality guidance, engagement in reflective practice, and raising concerns. The discussion will triangulate the results from different data sources (qualitative and quantitative) to understand the impact of the intervention using the Theory of Planned Behaviour (TPB) and position the study findings in the context of wider literature.

### Changes in professional behaviours

The findings reveal that the educational intervention significantly improved doctors’ attitudes, subjective norms, perceived behavioural control, and intention to refer to the *confidentiality guidance*. In the interviews participants expressed that the intervention helped to increase their knowledge of the guidance and confidence in applying it. New knowledge and confidence gained in turn increased their perceived behavioural control and intentions to use the guidance in the future. Doctors expressed positive attitudes towards the GMC’s confidentiality guidance even before the intervention and viewed the guidance as part of the law, with the guidance being acknowledged as something that everybody approved of (positive subjective norms). Nevertheless, participants’ attitudes and subjective norms improved after the intervention (changes in attitudes persisted after three-months) which shows that an educational intervention can significantly strengthen already positive attitudes/subjective norms towards certain professional behaviours.

DoaD participants expressed positive views towards *raising a concern*. However, this professional behaviour was perceived as complex and highly influenced by context. Quantitative analysis showed that attitudes, subjective norms, and perceived behavioural control towards raising a concern, but not intentions, improved in participants who attended the DoaD programme. In their interviews participants mentioned that the intervention empowered them to raise a concern which might have strengthened their positive views to raising a concern and feeling of control over this behaviour. However, these changes were not enough to increase participants’ intentions to engage in this complex behaviour. In interviews doctors expressed that raising a concern is challenging and mentioned numerous barriers to actually engage in such a behaviour, such as organisational culture, a lack of support, and potential difficulties between colleagues. Therefore, it seems that changes in cognition is not enough to engage in this context-dependent professional behaviour and changes in the environment need to be considered (e.g. organisational culture and support).

Minimal changes were observed after the intervention in the last professional behaviour, engagement in *reflective practice*. The quantitative data showed that the only TPB factor that improved significantly after the intervention was subjective norms, and this improvement persisted over time at three-months. Qualitative analysis revealed that the DoaD programme reinforced the benefits of reflection which might have highlighted a societal expectation that doctors should be reflective and, therefore, improved subjective norms. The quantitative data, however, showed no evidence of changes in other TBP factors; attitudes, perceived behavioural control, or intentions to reflect. Regarding attitudes and intentions to reflect, in the interviews participants spoke highly of reflection (positive attitudes), saying that they already engaged in it (strong intentions) before attending the programme. Even though in the interviews participants expressed that they will not reflect more after the intervention, it encouraged them to continue to engage in this behaviour. Regarding the lack of changes in perceived behaviour control, similarly to raising a concern, in interviews participants mentioned numerous barriers to being reflective (e.g. high demands). Differently from raising concerns, however, despite external barriers participants were already reflecting, demonstrating high personal control over the behaviour. What was still missing was control over external factors. Service delivery, working in unsupportive environments, and not having access to training or feedback on reflection – issues at the organisational level – were described as barriers to engage in reflective practice and would be difficult for individuals to change.

While there were no significant changes in the DoaD attendees’ attitudes towards reflection or intentions to reflect, there were differences between them and the control group participants: the control group’s attitudes and intentions around reflection became more negative over the data collection period. This may suggest that some influence external to the educational intervention took place that might have impacted the control group’s attitude and intentions toward reflection. However, the group that attended the programme did not show such negative changes, indicating that the programme acted as a protective measure against this negative influence. The quantitative data was collected during the widely discussed Dr. Bawa-Garba case, the trainee paediatrician who was removed from the UK medical register following the death of a child until winning an appeal. This case sparked controversy regarding reflective practice [21] conveying largely negative messages about reflection and might have affected participants’ views on reflective practice. However, it is not possible to say for certain if this event affected our study participants, as we did not conduct interviews during the quantitative data collection phase with control group participants, but it is clear from the data that attitudes and intentions remained stable (with non-significant increase) in the group attending the course, unlike the control group.

The paper by Page et al. (2020) [22] analysed the aspects of the education intervention which lead to changes in doctors’ attitudes and behavioural intentions. Using Persuasive Communication Theory they identified features pertaining to the educator, the content, and participants that were important in teaching professionalism and changing professional attitudes. The educator showed their credibility and trustworthiness, intervention’s content appealed rationally (e.g. data, logic argument) and emotionally (e.g. use of fear to promote compliance), and participants were shown discrepancy between currently held views and the educator’s message (e.g. GMC is a disciplinary body vs GMC supports doctors). The strategies through which changes were made are discussed in more detail in the Page et al. (2020) paper [22].

The study also revealed significant differences in the effectiveness of the educational intervention on UK and non-UK graduates. Specifically, the intervention had a significant effect in changing subjective norms towards raising concerns and using confidentiality guidance for non-UK graduates but not UK graduates. Non-UK graduates might be less influenced by the subjective norms of medical culture in the UK. Indeed, previous research suggests that there may be factors that influence specifically UK graduates’ perceptions of professional behaviour [23]. The attendance of the DoaD programme, therefore, may have had a stronger effect on non-UK graduates as their subjective norms were less strongly embedded compared to UK graduates who have longer experience in this culture. There are limited studies analysing differences in professional behaviours between UK and non-UK graduates, but the ones that do also show significant differences between these two groups. For example, one study revealed that non-UK graduates had more positive attitudes towards raising a concern, reflective practice, and the use of confidentiality guidance and that non-UK graduates also expressed stronger subjective norms towards the use of confidentiality guidance [11]. Studies also reveal that professionalism is contextual and, therefore, understood differently in different countries [24]. For example, faith might be an important background factor influencing professionalism in some countries (social accountability through accountably to God, divide accountability) [25].

### Positioning the study in the context of wider literature

Medical professionalism has been a subject of discussion among medical educators and researchers for decades with a growing interest in how to teach it. Teaching professionalism, however, is largely researched at undergraduate level and lacks the use of theoretical models (see systematic reviews [1, 26]). A recent systematic review on teaching professionalism in postgraduate training concluded that formal, structured teaching can improve professionalism in medical trainees but it also found that the majority of studies were conducted within one institution and just one fifth of eligible studies included controlled groups [27]. The current study overcomes these limitations and adds to existing knowledge by presenting the findings from a high quality mixed methods study evaluating an educational intervention based on the theoretical model and using data from geographically spread multiple locations. In addition, this study is unique in testing the effectiveness of an intervention for experienced doctors in the real world and investigating factors that might have an impact on its effectiveness. Professionalism is a social construction and cannot be considered to be stable [28] and with ever changing social and workplace environments, contextual factors in teaching professionalism should be considered. This study investigated not just if the intervention is effective but also for whom and why.

The current study found that an educational intervention improved TPB factors of three professional behaviours: raising a concern, engagement in reflective practice, and the use of confidentiality guidance. Despite a small second follow up sample, we found that some changes persisted three months after the intervention. Previous research also reveals that educational interventions in teaching professionalism can be successful [1, 26, 27]. Even though our study revealed that this educational intervention was successful in improving attitudes, subjective norms, perceived behaviour control and intentions, it is important to note that the effectiveness of the intervention depended on the complexity of the behaviour and participants’ characteristics. The intervention had the most positive effect on the use of confidentiality guidance: the strongest effect (the largest effect sizes) and improvement on all TPB factors (attitudes, subjective norms, perceived behaviour control, and intentions). It could be argued that the use of confidentiality guidance is a professional behaviour which is highly dependent on the level of knowledge (knowing about the guidance and its usefulness) and, therefore, is the easiest to teach in a formal education session. Other behaviours, however, are more difficult to change because they are more complex and context dependent. Some authors argue that professionalism can only be successfully learned if the medical educational institutions responsible for teaching it investigate and critically reflect upon their own professional culture and the values that are conveyed by their curriculum and the student body whom they admit [29]. Going beyond undergraduate education, the results of this study highlight that changes should be made in the workplace. For example, doctors expressed numerous barriers when talking about raising a concern. These barriers include disapproval from managers or other health care professionals, fears about the consequences of raising a concern on both themselves and others, the challenge of raising a concern about a person with greater authority and lack of feedback [16]. Some barriers are beyond the realms of an educational intervention and in order to change doctors’ professional behaviour, context level changes are required. We also noted that the effect of the intervention varied across different groups of doctors. Quantitative data revealed that subjective norms towards raising concerns and using confidentiality guidance significantly improved for non-UK graduates but not UK graduates. In our analysis we also controlled for participants’ years of experience and qualitative data showed that doctors had different views to professional behaviours depending on their experience. That is, senior doctors were more empowered to raise a concern following the intervention, while foundation doctors were more likely to raise an issue with their seniors. These findings highlight that the same intervention can have a different effect on participants depending on their characteristics. This is an important finding showing that more tailored interventions or strategies might need to be applied in teaching professionalism.

## Strengths and limitations

The study investigates the complex topic of teaching professionalism using a theoretical model and mixed methods design combining qualitative interview data and quasi-experimental design survey. Another strength of this study is that unlike the majority of studies in this area [27], data were collected from multiple geographically spread locations and participants from a variety of backgrounds, specialty areas, and levels of experience.

This study did not, however, measure behaviours directly and future research should include behaviour measures in their evaluation. Future studies should also develop interventions based on the theoretical model as this study applied the theory to the evaluation only. Another limitation of this study is the small number of participants who took part in the Time-3 survey and no control group comparison at this time point. A further limitation of this study is that we were not able to calculate a response rate for the questionnaire because of a third party (i.e., NHS Trusts) being responsible for data collection on our behalf and we were not able to obtain precise data on the numbers of doctors invited to take part in the study. We have invited doctors to fill in the Time-2 questionnaire 30 days after the intervention, but this principle was not strictly followed and some participated slightly later.

## Conclusion

This study used a theoretical framework (TPB) to investigate the effectiveness of an educational intervention in improving three professional behaviours: use of confidentiality guidance, reflective practice, and raising concerns. The study revealed that the intervention improved doctors’ attitudes, social norms, perceived behaviour control, and intentions to engage in these three professional behaviours. Despite the positive results, this study also showed that teaching professionalism for practicing doctors is complex and the effectiveness of an educational intervention depends on the environmental context and personal factors. The study found that the effectiveness of the intervention was different for UK and non-UK graduates, who have different prior experiences of professional behaviour in the UK. In addition, in order to change more complex professional behaviour, such as raising concerns, changes in cognition alone are not sufficient. That is, barriers experienced at the organisational and system level should additionally be addressed.

## Supplementary Information

**Additional file 1.**

**Additional file 2.**

**Additional file 3.**

**Additional file 4.**

**Additional file 5.**

**Additional file 6.**

## Data Availability

The data generated and analysed during the current study are not available as consent for this has not been granted by participants.

## Abbreviations

DoaD programme: GMC’s Duties of a Doctor programme
BME: Black and minority ethnic
GMC: General Medical Council
HEE: Higher Education England
LRMP: List of Registered Medical Practitioners
RLA: Regional Liaison Advisors
RLS: GMC’s Regional Liaison Service
SAS: Specialty and Associate Specialist
TPB: Theory of Planned Behaviour
PMQ: Primary medical qualification
