# Supplementary material for: Changing professional behaviours: mixed methods study utilising psychological theories to evaluate an educational programme for UK medical doctors
Source: BMC Med Educ. 2021 Feb 5;21:92. doi: 10.1186/s12909-021-02510-4 (PMC7866444; doi:10.1186/s12909-021-02510-4)
Supplement: Supplementary file 1 — Additional file 1. [file 12909_2021_2510_MOESM1_ESM.docx]

## **Exploring Doctors’ Professional Behaviours**

## **Raising Concerns**

The following questions are about raising a concern at work. We would like to know what you think about this, regardless of whether or not you have had/seen a significant issue at work e.g. a patient safety concern.

1. **Overall, I think that raising a concern is:** *(please circle)*

| *Worthless* | 1 | 2 | 3 | 4 | 5 | 6 | 7 | *Worthwhile* |
| --- | --- | --- | --- | --- | --- | --- | --- | --- |
| *Unpleasant* | 1 | 2 | 3 | 4 | 5 | 6 | 7 | *Pleasant* |
| *Difficult* | 1 | 2 | 3 | 4 | 5 | 6 | 7 | *Easy* |
| *The wrong thing to do* | 1 | 2 | 3 | 4 | 5 | 6 | 7 | *The right thing to do* |
| *Bad practice* | 1 | 2 | 3 | 4 | 5 | 6 | 7 | *Best practice* |

1. **Please rate how much you personally agree or disagree with the statements below:** *(please circle)*

|  | *Strongly disagree* | |  |  |  | *Strongly agree* | |
| --- | --- | --- | --- | --- | --- | --- | --- |
| i. I would feel comfortable speaking to the GMC if I had a concern at work | 1 | 2 | 3 | 4 | 5 | 6 | 7 |
| ii. People who are important to me think  I should not report a concern if I have one *(R)* | 1 | 2 | 3 | 4 | 5 | 6 | 7 |
| iii. I plan to raise a concern if I have one in my workplace | 1 | 2 | 3 | 4 | 5 | 6 | 7 |
| iv. I don’t intend to raise a concern if I have one in my workplace *(R)* | 1 | 2 | 3 | 4 | 5 | 6 | 7 |
| v. It is expected of me that I report a concern if I have one | 1 | 2 | 3 | 4 | 5 | 6 | 7 |
| vi. I am confident that I can raise a concern if I want to | 1 | 2 | 3 | 4 | 5 | 6 | 7 |
| vii. I want to raise a concern when I have one in my work environment | 1 | 2 | 3 | 4 | 5 | 6 | 7 |

1. **Please indicate how much pressure you would feel from each of the following organisations or people to raise a concern if you had one** *(please circle)***:**

|  | *No pressure* | |  |  | *Strong pressure* | | |
| --- | --- | --- | --- | --- | --- | --- | --- |
| i. The GMC | 1 | 2 | 3 | 4 | 5 | 6 | 7 |
| ii. My Trust/My Practice | 1 | 2 | 3 | 4 | 5 | 6 | 7 |
| iii. Medical Defence Organisation | 1 | 2 | 3 | 4 | 5 | 6 | 7 |
| iv. Senior colleagues | 1 | 2 | 3 | 4 | 5 | 6 | 7 |
| v. Peers | 1 | 2 | 3 | 4 | 5 | 6 | 7 |
| vi. My appraiser | 1 | 2 | 3 | 4 | 5 | 6 | 7 |
| vii. Patients | 1 | 2 | 3 | 4 | 5 | 6 | 7 |
| viii. The media | 1 | 2 | 3 | 4 | 5 | 6 | 7 |
| ix. Healthcare professionals | 1 | 2 | 3 | 4 | 5 | 6 | 7 |

1. **For me to report a patient safety concern is:** *(please circle)*

| *Difficult* | 1 | 2 | 3 | 4 | 5 | 6 | 7 | *Easy* |
| --- | --- | --- | --- | --- | --- | --- | --- | --- |

Attitudes: 1.1-1.5 + 2.1;

Subjective norms: 2.2 + 2.5 + 3.1-3.9;

Perceived behaviours control: 2.6 + 4;

Intentions: 2.3 + 2.4 + 2.7

## **Section Four: Reflective Practice**

1. **Overall, I think that reflective practice is:** *(please circle)*

| *Worthless* | 1 | 2 | 3 | 4 | 5 | 6 | 7 | *Worthwhile* |
| --- | --- | --- | --- | --- | --- | --- | --- | --- |
| *Difficult* | 1 | 2 | 3 | 4 | 5 | 6 | 7 | *Easy* |
| *Irrelevant* | 1 | 2 | 3 | 4 | 5 | 6 | 7 | *Relevant* |
| *Unpleasant (for me)* | 1 | 2 | 3 | 4 | 5 | 6 | 7 | *Pleasant (for me)* |
| *The wrong thing to do* | 1 | 2 | 3 | 4 | 5 | 6 | 7 | *The right thing to do* |
| *Bad practice* | 1 | 2 | 3 | 4 | 5 | 6 | 7 | *Best practice* |

1. **Please rate how much you personally agree or disagree with the statements below:** *(please circle)*

|  | *Strongly disagree* | |  |  |  | *Strongly agree* | |
| --- | --- | --- | --- | --- | --- | --- | --- |
| i. Reflecting on my practice makes me a better doctor | 1 | 2 | 3 | 4 | 5 | 6 | 7 |
| ii. Reflecting on my practice demonstrates that I am trying to become a better doctor | 1 | 2 | 3 | 4 | 5 | 6 | 7 |
| iii. I don’t plan to reflect on my practice *(R)* | 1 | 2 | 3 | 4 | 5 | 6 | 7 |
| iv. People who are important to me think  I should reflect on my practice | 1 | 2 | 3 | 4 | 5 | 6 | 7 |
| v. I intend to reflect on my practice | 1 | 2 | 3 | 4 | 5 | 6 | 7 |
| vi. It is expected of me that I reflect on my practice | 1 | 2 | 3 | 4 | 5 | 6 | 7 |
| vii. I want to reflect on my practice | 1 | 2 | 3 | 4 | 5 | 6 | 7 |

1. **Please indicate how much pressure you feel from each of the following organisations or people to reflect on your practice:** *(please circle)*

|  | *No pressure* | |  |  | *Strong pressure* | | |
| --- | --- | --- | --- | --- | --- | --- | --- |
| i. The GMC | 1 | 2 | 3 | 4 | 5 | 6 | 7 |
| ii. My Trust/ My Practice | 1 | 2 | 3 | 4 | 5 | 6 | 7 |
| iii. Medical Defence Organisation | 1 | 2 | 3 | 4 | 5 | 6 | 7 |
| iv. Senior colleagues | 1 | 2 | 3 | 4 | 5 | 6 | 7 |
| v. Peers | 1 | 2 | 3 | 4 | 5 | 6 | 7 |
| vi. Responsible Officer | 1 | 2 | 3 | 4 | 5 | 6 | 7 |
| vii. My appraiser | 1 | 2 | 3 | 4 | 5 | 6 | 7 |
| viii. Patients | 1 | 2 | 3 | 4 | 5 | 6 | 7 |
| ix. The media | 1 | 2 | 3 | 4 | 5 | 6 | 7 |
| x. Healthcare professionals | 1 | 2 | 3 | 4 | 5 | 6 | 7 |

1. **For me to reflect on my practice is:** *(please circle)*

| *Difficult* | 1 | 2 | 3 | 4 | 5 | 6 | 7 | *Easy* |
| --- | --- | --- | --- | --- | --- | --- | --- | --- |

Attitudes: 5.1-5.6 + 6.1 + 6.2;

Subjective norms: 6.4 + 6.6 + 7.1-7.10;

Perceived behaviours control: 8;

Intentions: 6.3 + 6.5 + 6.7

## **Confidentiality**

The following questions are about the GMC confidentiality guidance.

1. **Overall, I think that the GMC confidentiality guidance is:** *(please circle)*

| *Hard to access* | 1 | 2 | 3 | 4 | 5 | 6 | 7 | *Easy to access* |
| --- | --- | --- | --- | --- | --- | --- | --- | --- |
| *Hard to apply* | 1 | 2 | 3 | 4 | 5 | 6 | 7 | *Easy to apply* |
| *Unrealistic* | 1 | 2 | 3 | 4 | 5 | 6 | 7 | *Realistic* |
| *Unclear* | 1 | 2 | 3 | 4 | 5 | 6 | 7 | *Clear* |
| *Harmful* | 1 | 2 | 3 | 4 | 5 | 6 | 7 | *Beneficial* |
| *Worthless* | 1 | 2 | 3 | 4 | 5 | 6 | 7 | *Worthwhile* |

1. **Please rate how much you personally agree or disagree with the statements below:** *(please circle)*

|  | *Strongly disagree* | |  |  |  | *Strongly agree* | |
| --- | --- | --- | --- | --- | --- | --- | --- |
| i. People who are important to me think I should use the GMC confidentiality guidance | 1 | 2 | 3 | 4 | 5 | 6 | 7 |
| ii. It is expected of me to use the GMC confidentiality guidance | 1 | 2 | 3 | 4 | 5 | 6 | 7 |
| iii. I am more likely to speak to colleagues for guidance on confidentiality than use the GMC guidance *(R)* | 1 | 2 | 3 | 4 | 5 | 6 | 7 |
| iv. I am more likely to speak to my Medical Defence Organisation for guidance on confidentiality than the GMC *(R)* | 1 | 2 | 3 | 4 | 5 | 6 | 7 |
| v. I am confident that I cannot apply the GMC confidentiality guidance in practice *(R)* | 1 | 2 | 3 | 4 | 5 | 6 | 7 |
| vi. I have enough time to refer to the GMC confidentiality guidance | 1 | 2 | 3 | 4 | 5 | 6 | 7 |
| vii. I can easily navigate the GMC website to check confidentiality guidelines | 1 | 2 | 3 | 4 | 5 | 6 | 7 |
| viii. I intend to refer to the GMC confidentiality guidance the next time I’m uncertain | 1 | 2 | 3 | 4 | 5 | 6 | 7 |
| ix. I want to use the GMC confidentiality guidance | 1 | 2 | 3 | 4 | 5 | 6 | 7 |
| x. I don’t plan to use the GMC confidentiality guidance *(R)* | 1 | 2 | 3 | 4 | 5 | 6 | 7 |

**11.Please indicate how much pressure you feel from each of the following organisations or people to use the GMC’s confidentiality guidance:** *(please circle)*

|  | *No pressure* | |  |  | *Strong pressure* | | |
| --- | --- | --- | --- | --- | --- | --- | --- |
| i. The GMC | 1 | 2 | 3 | 4 | 5 | 6 | 7 |
| ii. My Trust/ My Practice | 1 | 2 | 3 | 4 | 5 | 6 | 7 |
| iii. Medical Defence Organisation | 1 | 2 | 3 | 4 | 5 | 6 | 7 |
| iv. Senior colleagues | 1 | 2 | 3 | 4 | 5 | 6 | 7 |
| v. Peers | 1 | 2 | 3 | 4 | 5 | 6 | 7 |
| vi. My appraiser | 1 | 2 | 3 | 4 | 5 | 6 | 7 |
| vii. Patients | 1 | 2 | 3 | 4 | 5 | 6 | 7 |
| viii. The media | 1 | 2 | 3 | 4 | 5 | 6 | 7 |
| ix. Healthcare professionals | 1 | 2 | 3 | 4 | 5 | 6 | 7 |

**12.For me to apply the GMC confidentiality guidance in my practice is:** *(please circle)*

| *Difficult* | 1 | 2 | 3 | 4 | 5 | 6 | 7 | *Easy* |
| --- | --- | --- | --- | --- | --- | --- | --- | --- |

Attitudes: 9.1-9.6 + 9.3 + 9.4;

Subjective norms: 9.1 + 9.2 + 11.1-11.9;

Perceived behaviours control: 9.5 + 9.6 + 9.7 + 12;

Intentions: 9.8-9.10
