# Supplementary material for: Changing professional behaviours: mixed methods study utilising psychological theories to evaluate an educational programme for UK medical doctors
Source: BMC Med Educ. 2021 Feb 5;21:92. doi: 10.1186/s12909-021-02510-4 (PMC7866444; doi:10.1186/s12909-021-02510-4)
Supplement: Supplementary file 2 — Additional file 2. [file 12909_2021_2510_MOESM2_ESM.docx]

# Supplementary File 1

*Table 1*. Differences in theory of planned behaviour factors of three professional behaviours at baseline (Time-1)

| Scale | | M (*SD*) | | | Differences between groups |
| --- | --- | --- | --- | --- | --- |
|  |  | **Total** | **Control group (n=94)** | **Intervention group (n=111)** |  |
| Raising concerns | Attitudes | 4.12 (1.09) | 3.90 (0.92) | 4.31 (1.18) | ***t*(201.76) = -2.751, *p* = .006** |
|  | Subjective norms | 4.71 (1.03) | 4.72 (1.06) | 4.71 (1.00) | *t*(203) = 0.034, *p* = .973 |
|  | Perceived behaviour control | 5.19 (1.31) | 5.23 (1.28) | 5.15 (1.33) | *t*(203) = 0.412, *p* = .681 |
|  | Intentions | 5.56 (1.12) | 5.63 (1.18) | 5.50 (1.07) | *t*(203) = 0.827, *p* = .409 |
| Reflective practice | Attitudes | 5.26 (1.16) | 5.14 (1.17) | 5.35 (1.15) | *t*(203) = -1.295, *p* = .197 |
|  | Subjective norms | 4.57 (1.09) | 4.49 (1.04) | 4.64 (1.13) | *t*(203) = -0.964, *p* = .336 |
|  | Perceived behaviour control | 4.98 (1.54) | 4.94 (1.68) | 5.02 (1.42)^1^ | *t*(202) = -0.377, *p* = .706 |
|  | Intentions | 6.00 (1.09) | 5.88 (1.17) | 6.10 (1.01) | *t*(203) = -1.493, *p* = .137 |
| Use of confidentiality guidance | Attitudes | 4.60 (1.10) | 4.67 (1.09) | 4.55 (1.11) ^1^ | *t*(202) = 0.768, *p* = .443 |
|  | Subjective norms | 4.20 (1.46) | 4.06 (1.54) | 4.32 (1.39) ^2^ | *t*(201) = -1.282, *p* = .201 |
|  | Perceived behaviour control | 5.23 (1.14) | 4.45 (1.16) | 4.53 (1.12) ^2^ | *t*(201) = -0.497, *p* = .620 |
|  | Intentions | 4.50 (1.28) | 4.98 (1.36) | 5.44 (1.17) ^1^ | ***t*(202) = -2.630, *p* = .009** |

*Note*. ^1^ n=110; ^2^ n=109
