# Supplementary material for: Changing professional behaviours: mixed methods study utilising psychological theories to evaluate an educational programme for UK medical doctors
Source: BMC Med Educ. 2021 Feb 5;21:92. doi: 10.1186/s12909-021-02510-4 (PMC7866444; doi:10.1186/s12909-021-02510-4)
Supplement: Supplementary file 3 — Additional file 3. [file 12909_2021_2510_MOESM3_ESM.docx]

# Supplementary File 4

*Table 1*. Demographic characteristics of interview participants

| **Location alias** | **Ethnicity** | **Group** | **UK/non-UK graduates** | **Gender** | **Topic covered** |
| --- | --- | --- | --- | --- | --- |
| 1 | White | Established consultants/ST6 | UK | Male | Reflection and confidentiality |
| 1 | White British | Established consultants/ST6 | UK | Female | Confidentiality and reflection |
| 1 | White | Consultant/SAS doctors | UK | Female | Reflection |
| 2 | Asian | SAS doctors | Non-UK | Male | Confidentiality |
| 2 | Asian | SAS doctors | Non-UK | Male | Confidentiality |
| 2 | Asian | SAS doctors | Non-UK | Male | Confidentiality |
| 3 | Not Given | FY2 doctors | UK | Female | Confidentiality |
| 3 | White British | FY2 doctors | UK | Male | Confidentiality |
| 3 | White British | FY2 doctors | UK | Male | confidentiality |
| 3 | Asian | FY2 doctors | UK | Male | Confidentiality |
| 3 | Not Given | FY2 doctors | UK | Male | Reflection |
| 3 | Asian | FY2 doctors | UK | Female | Reflection |
| 3 | Not Given | FY2 doctors | UK | Male | Reflection |
| 3 | White British | FY2 doctors | UK | Female | Reflection |
| 3 | White British | FY2 doctors | UK | Male | Raising concerns |
| 3 | White British | FY2 doctors | UK | Male | Raising concerns |
| 3 | White British | FY2 doctors | UK | Male | Raising concerns |
| 3 | Asian British | FY2 doctors | UK | Female | Raising concerns |
| 3 | White British | FY2 doctors | UK | Female | Raising concerns |
| 3 | Not Given | FY2 doctors | UK | Female | Raising concerns |
| 3 | Not Given | FY2 doctors | UK | Female | Raising concerns |
| 3 | White British | FY2 doctors | UK | Female | Raising concerns |
| 4 | Asian British | New consultants | Non-UK | Male | Reflection |
| 4 | Asian British | New consultants | UK | Female | Reflection |
| 4 | Asian | New consultants | UK | Female | Reflection |
| 4 | White | New consultants | Non-UK | Female | Reflection |
| 5 | White | Consultant/SAS doctors | UK | Female | Raising concerns |
| 5 | White British | Consultant/SAS doctors | UK | Female | Raising concerns |
| 5 | White British | Consultant/SAS doctors | UK | Female | Raising concerns |
| 5 | Asian | Consultant/SAS doctors | Non-UK | Male | Raising concerns |
| 5 | White | Consultant/SAS doctors | Non-UK | Female | Reflection and raising concerns |
| 5 | White British | Consultant/SAS doctors | UK | Male | Raising concerns |
| 5 | White | Consultant/SAS doctors | UK | Male | Raising concerns |
| 5 | Asian | Consultant/SAS doctors | Non-UK | Female | Raising concerns |
| 6 | White | Non-UK | Non-UK | Male | Reflection |
| 6 | Asian | Non-UK | Non-UK | Male | Reflection |
| 6 | Other | Non-UK | Non-UK | Male | Reflection |
| 7 | White British | GPs/Consultants | UK | Male | Reflection |
| 7 | Not Given | GPs/Consultants | UK | Male | Reflection |
| 7 | Not Given | GPs/Consultants | UK | Female | Reflection |
| 7 | White | GPs/Consultants | UK | Male | Raising concerns and confidentiality |
| 7 | Not Given | GPs/Consultants | UK | Female | Confidentiality |
