# Supplementary material for: Changing professional behaviours: mixed methods study utilising psychological theories to evaluate an educational programme for UK medical doctors
Source: BMC Med Educ. 2021 Feb 5;21:92. doi: 10.1186/s12909-021-02510-4 (PMC7866444; doi:10.1186/s12909-021-02510-4)
Supplement: Supplementary file 4 — Additional file 4. [file 12909_2021_2510_MOESM4_ESM.docx]

# Supplementary File 3

*Figure 1*. Participant flow diagram
