# Supplementary material for: Changing professional behaviours: mixed methods study utilising psychological theories to evaluate an educational programme for UK medical doctors
Source: BMC Med Educ. 2021 Feb 5;21:92. doi: 10.1186/s12909-021-02510-4 (PMC7866444; doi:10.1186/s12909-021-02510-4)
Supplement: Supplementary file 5 — Additional file 5. [file 12909_2021_2510_MOESM5_ESM.docx]

# Supplementary File 4

*Table 1*. Results from the spit-plot ANCOVA analysis

| **Group** | **Time point** | **Marginal Mean scores** | **Marginal Mean difference (T-2 - T-1)** | **PMQ** | **Marginal Mean scores** | **Marginal Mean difference (Intervention-control)** | **Marginal Mean difference (T2 – T1)** | **Covariate effect of # of years of experience** | **Main effect of PMQ** | **Main effect of Group** | **Interaction effect of Group X Time** | **Interaction effect of Group x Time x PMQ** |  |
| --- | --- | --- | --- | --- | --- | --- | --- | --- | --- | --- | --- | --- | --- |
| Raising concerns – Attitudes | | | | | | | | | | | | | |
| Control | Time-1 | 3.97 | -0.079 (*F* = 0.280, *p* = .597, ηp^2^ = .001) | UK | 3.98 | ***UK T-1****:* 0.047 (*F* = 0.061, *p* = .806, ηp^2^ < .001)  ***UK T-2:* 0.655 (*F* = 12.947, *p* < .001, ηp^2^ = .061)**  ***Non-UK T-1****:* 0.494 (*F* = 2.262, *p* = .134, ηp^2^ = .011)  ***Non-UK T-2:*** **0.720 (*F* = 5.204, *p* = .024, ηp^2^ = .026)** | ***UK****:* ***-*0.246 (*F* = 4.035, *p* = .046, ηp^2^ = .020)**  ***Non-UK:*** 0.089 (*F* = 0.108, *p* = .742, ηp^2^ = .001) | *F*(1,198) = 4.948, *p* = .080, ηp^2^ = .015 | *F*(1,198) = 3.000, *p* = .085, ηp^2^ = .015 | ***F*(1,198) = 8.300, *p* = .004, ηp^2^ = .040** | ***F*(1,198) = 4.991, *p* = .027, ηp^2^ = .025** | *F*(1,198) = 1.153, *p* = .284, ηp^2^ = .006 |  |
|  |  |  |  | Non-UK | 3.97 |  |  |  |  |  |  |  |  |
|  | Time-2 | 3.89 |  | UK | 3.73 |  |  |  |  |  |  |  |  |
|  |  |  |  | Non-UK | 4.06 |  |  |  |  |  |  |  |  |
| Intervention | Time-1 | 4.24 | **0.338 (*F* = 10.905, *p* = .001, ηp^2^ = .052)** | UK | 4.02 |  | ***UK****:* **0.362 (*F* = 6.520, *p* = .011, ηp^2^ = .032)**  ***Non-UK:* 0.315 (*F* = 4.000, *p* = .047, ηp^2^ = .020)** |  |  |  |  |  |  |
|  |  |  |  | Non-UK | 4.46 |  |  |  |  |  |  |  |  |
|  | Time-2 | 4.58 |  | UK | 4.38 |  |  |  |  |  |  |  |  |
|  |  |  |  | Non-UK | 4.78 |  |  |  |  |  |  |  |  |
| Raising concerns – Subjective norms | | | | | | | | | | | | | |
| Control | Time-1 | 4.89 | -0.174 (*F* = 1.506, *p* = .221, ηp^2^ = .008) | UK | 4.74 | ***UK T-1****:* 0.107 (*F* = 0.324, *p* = .570, ηp^2^ = .002)  ***UK T-2:*** 0.020 (*F* = 0.011, *p* = .916, ηp^2^ < .001)  ***Non-UK T-1****: -*0.561 (*F* = 3.015, *p* = .084, ηp^2^ = .015)  ***Non-UK T-2:*** 0.338 (*F* = 1.109, *p* = .294, ηp^2^ = .006) | ***UK****:* 0.167 (*F* = 2.068, *p* = .152, ηp^2^ = .010)  ***Non-UK:*** **-0.515 (*F* = 4.059, *p* = .045, ηp^2^ = .020)** | *F*(1,198) = 2.461, *p* = .118, ηp^2^ = .012 | *F*(1,198) = 0.528, *p* = .468, ηp^2^ = .003 | *F*(1,198) = 0.021, *p* = .886, ηp^2^ < .001 | ***F*(1,198) = 5.238, *p* = .023, ηp^2^ = .026** | ***F*(1,198) = 8.510, *p* = .004, ηp^2^ = .041** |  |
|  |  |  |  | Non-UK | 5.04 |  |  |  |  |  |  |  |  |
|  | Time-2 | 4.71 |  | UK | 4.90 |  |  |  |  |  |  |  |  |
|  |  |  |  | Non-UK | 4.53 |  |  |  |  |  |  |  |  |
| Intervention | Time-1 | 4.66 | **0.232 (*F* = 5.696, *p* = .018, ηp^2^ = .028)** | UK | 4.84 |  | ***UK****:* 0.080 (*F* = 0.357, *p* = .551, ηp^2^ = .002)  ***Non-UK:* 0.384 (*F* = 6.602, *p* = .011, ηp^2^ = .032)** |  |  |  |  |  |  |
|  |  |  |  | Non-UK | 4.48 |  |  |  |  |  |  |  |  |
|  | Time-2 | 4.89 |  | UK | 4.92 |  |  |  |  |  |  |  |  |
|  |  |  |  | Non-UK | 4.86 |  |  |  |  |  |  |  |  |
| Raising concerns – Perceived behaviour control | | | | | | | | | | | | | |
| Control | Time-1 | 5.35 | -0.115 (*F* = 0.391, *p* = .532, ηp^2^ = .002) | UK | 5.19 | ***UK T-1****:-* 0.079 (*F* = 0.109, *p* = .742, ηp^2^ = .001)  ***UK T-2:*** -0.368 (*F* = 2.635, *p* = .106, ηp^2^ = .013)  ***Non-UK T-1****: -*0.320 (*F* = 0.594, *p* = .442, ηp^2^ = .003)  ***Non-UK T-2:*** 0.157 (*F* = 0.161, *p* = .688, ηp^2^ = .001) | ***UK****: -*0.227 (*F* = 2.230, *p* = .137, ηp^2^ = .011)  ***Non-UK:*** -0.004 (*F* < 0.001, *p* = .990, ηp^2^ < .001) | F(1,198) = 0.001, *p* = .971, ηp2 < .001 | *F*(1,198) = 2.191, *p* = .140, ηp^2^ = .011 | *F*(1,198) = 0.023, *p* = .881, ηp^2^ < .001 | ***F*(1,198) = 3.996, *p* = .047, ηp^2^ = .020** | *F*(1,198) = 0.005, *p* = .946, ηp^2^ < .001 |  |
|  |  |  |  | Non-UK | 5.50 |  |  |  |  |  |  |  |  |
|  | Time-2 | 5.23 |  | UK | 4.96 |  |  |  |  |  |  |  |  |
|  |  |  |  | Non-UK | 5.50 |  |  |  |  |  |  |  |  |
| Intervention | Time-1 | 5.15 | **0.347 (*F* = 7.475, *p* = .007, ηp^2^ = .036)** | UK | 5.11 |  | ***UK****:* 0.221 (*F* = 1.580, *p* = .210, ηp^2^ = .008)  ***Non-UK:*** **0.473 (*F* = 5.895, *p* = .016, ηp^2^ = .029)** |  |  |  |  |  |  |
|  |  |  |  | Non-UK | 5.18 |  |  |  |  |  |  |  |  |
|  | Time-2 | 5.50 |  | UK | 5.33 |  |  |  |  |  |  |  |  |
|  |  |  |  | Non-UK | 5.66 |  |  |  |  |  |  |  |  |
| Raising concerns – Intentions | | | | | | | | | | | | | |
| Control | Time-1 | 5.72 | -0.139 (*F* = 0.696, *p* = .405, ηp^2^ = .004) | UK | 5.59 | ***UK T-1****: -*0.070 (*F* = 0.116, *p* = .734, ηp^2^ = .001)  ***UK T-2:*** 0.167 (*F* = 0.725, *p* = 0.396, ηp^2^ = .004)  ***Non-UK T-1****: -*0.355 (*F* = 0.993, *p* = .320, ηp^2^ = .005)  ***Non-UK T-2:*** 0.154 (*F* = 0.207, *p* = .650, ηp^2^ = .001) | ***UK****: -*0.050 (*F* = 0.132, *p* = .717, ηp^2^ = .001)  ***Non-UK:*** -0.228 (*F* = 0.576, *p* = .449, ηp^2^ = .003) | F(1,198) = 0.075, *p* = .784, ηp^2^ < .001 | *F*(1,198) = 0.318, *p* = .573, ηp^2^ = .002 | *F*(1,198) = 0.021, *p* = .884, ηp^2^ < .001 | *F*(1,198) = 3.190, *p* = .076, ηp^2^ = .016 | *F*(1,198) = 0.464, *p* = .497, ηp^2^ = .002 |  |
|  |  |  |  | Non-UK | 5.86 |  |  |  |  |  |  |  |  |
|  | Time-2 | 5.59 |  | UK | 5.54 |  |  |  |  |  |  |  |  |
|  |  |  |  | Non-UK | 5.63 |  |  |  |  |  |  |  |  |
| Intervention | Time-1 | 5.51 | **0.234 (*F* = 4.170, *p* = .042, ηp^2^ = .021)** | UK | 5.52 |  | ***UK****:* 0.188 (*F* = 1.403, *p* = .238, ηp^2^ = .007)  ***Non-UK:*** 0.280 (*F* = 2.535, *p* = .113, ηp^2^ = .013) |  |  |  |  |  |  |
|  |  |  |  | Non-UK | 5.50 |  |  |  |  |  |  |  |  |
|  | Time-2 | 5.75 |  | UK | 5.71 |  |  |  |  |  |  |  |  |
|  |  |  |  | Non-UK | 5.78 |  |  |  |  |  |  |  |  |
| Reflection – Attitudes | | | | | | | | | | | | | |
| Control | Time-1 | 5.25 | -0.270 (*F* = 3.864, *p* = .051, ηp^2^ = .019) | UK | 5.08 | ***UK T-1****:* 0.087 (*F* = 0.175, *p* = .676, ηp^2^ = .001)  ***UK T-2:* 0.497 (*F* = 2.635, *p* = .017, ηp^2^ = .013)**  ***Non-UK T-1****: -*0.084 (*F* = 0.054, *p* = .816, ηp^2^ < .001)  ***Non-UK T-2:*** 0.277 (*F* = 0.608, *p* = .436, ηp^2^ = .003) | ***UK****: -*0.134 (*F* = 1.403, *p* = .238, ηp^2^ = .007)  ***Non-UK:*** -0.407 (*F* = 2.681, *p* = .103, ηp^2^ = .013) | F(1,198) = 1.346, *p* = .247, ηp^2^ = .007 | *F*(1,198) = 2.344, *p* = .127, ηp^2^ = .012 | *F*(1,198) = 1.012, *p* = .316, ηp^2^ = .005 | ***F*(1,198) = 5.005, *p* = .026, ηp^2^ = .025** | *F*(1,198) = 0.022, *p* = .882, ηp^2^ < .001 |  |
|  |  |  |  | Non-UK | 5.61 |  |  |  |  |  |  |  |  |
|  | Time-2 | 5.08 |  | UK | 4.95 |  |  |  |  |  |  |  |  |
|  |  |  |  | Non-UK | 5.21 |  |  |  |  |  |  |  |  |
| Intervention | Time-1 | 5.35 | 0.115 (*F* = 1.485, *p* = .224, ηp^2^ = .007) | UK | 5.17 |  | ***UK****:* **0.276 (*F* = 4.449, *p* = .036, ηp^2^ = .022)**  ***Non-UK: -***0.045 (*F* = 0.098, *p* = .755, ηp^2^ < .001) |  |  |  |  |  |  |
|  |  |  |  | Non-UK | 5.53 |  |  |  |  |  |  |  |  |
|  | Time-2 | 5.47 |  | UK | 5.45 |  |  |  |  |  |  |  |  |
|  |  |  |  | Non-UK | 5.48 |  |  |  |  |  |  |  |  |
| Reflection – Subjective norms | | | | | | | | | | | | | |
| Control | Time-1 | 4.45 | -0.073 (*F* = 0.264, *p* = .608, ηp^2^ = .001) | UK | 4.47 | ***UK T-1****:* 0.185 (*F* = 0.861, *p* = .355, ηp^2^ = .004)  ***UK T-2:* 0.439 (*F* = 4.847, *p* = .029, ηp^2^ = .024)**  ***Non-UK T-1****:* 0.264 (*F* = 0.587, *p* = .444, ηp^2^ = .003)  ***Non-UK T-2:*** **0.912 (*F* = 7.033, *p* = .009, ηp^2^ = .034)** | ***UK****:* 0.048 (*F* = 0.171, *p* = .680, ηp^2^ = .001)  ***Non-UK:*** -0.194 (*F* = 0.574, *p* = .450, ηp^2^ = .003) | F(1,198) = 0.409, *p* = .523, ηp^2^ = .002 | *F*(1,198) = 0.024, *p* = .877, ηp^2^ < .001 | ***F*(1,198) = 6.034, *p* = .015, ηp^2^ = .030** | ***F*(1,198) = 6.410, *p* = .012, ηp^2^ = .031** | *F*(1,198) = 1.351, *p* = .247, ηp^2^ = .007 |  |
|  |  |  |  | Non-UK | 4.43 |  |  |  |  |  |  |  |  |
|  | Time-2 | 4.37 |  | UK | 4.52 |  |  |  |  |  |  |  |  |
|  |  |  |  | Non-UK | 4.23 |  |  |  |  |  |  |  |  |
| Intervention | Time-1 | 4.67 | **0.378 (*F* = 14.953, *p* < .001, ηp^2^ = .070)** | UK | 4.66 |  | ***UK****:* **0.302 (*F* = 4.991, *p* = .027, ηp^2^ = .025)**  ***Non-UK:* 0.453 (*F* = 9.140, *p* = .003, ηp^2^ = .044)** |  |  |  |  |  |  |
|  |  |  |  | Non-UK | 4.69 |  |  |  |  |  |  |  |  |
|  | Time-2 | 5.05 |  | UK | 4.96 |  |  |  |  |  |  |  |  |
|  |  |  |  | Non-UK | 5.14 |  |  |  |  |  |  |  |  |
| Reflection – Perceived behaviour control | | | | | | | | | | | | | |
| Control | Time-1 | 5.06 | -0.056 (*F* = 0.057, *p* = .811, ηp^2^ < .001) | UK | 4.89 | ***UK T-1****:* -0.011 (*F* = 0.002, *p* = .969, ηp^2^ < .001)  ***UK T-2:*** 0.023 (*F* = 0.006, *p* = .938, ηp^2^ = .024)  ***Non-UK T-1****:*  -0.086 (*F* = 0.031, *p* = .861, ηp^2^ < .001)  ***Non-UK T-2:*** 0.129 (*F* = 0.068, *p* = .794, ηp^2^ < .001) | ***UK****: -*0.170 (*F* = 0.793, *p* = .374, ηp^2^ = .004)  ***Non-UK:*** 0.059 (*F* = 0.020, *p* = .888, ηp^2^ < .001) | F(1,196) = 0.068, *p* = .795, ηp^2^ < .001 | *F*(1,196) = 3.068, *p* = .081, ηp^2^ = .015 | *F*(1,196) = 0.003, *p* = .957, ηp^2^ < .001 | *F*(1,196) = 0.181, *p* = .671, ηp^2^ = .001 | *F*(1,196) = 0.106, *p* = .746, ηp^2^ = .001 |  |
|  |  |  |  | Non-UK | 5.22 |  |  |  |  |  |  |  |  |
|  | Time-2 | 5.00 |  | UK | 4.72 |  |  |  |  |  |  |  |  |
|  |  |  |  | Non-UK | 5.28 |  |  |  |  |  |  |  |  |
| Intervention | Time-1 | 5.01 | 0.069 (*F* = 0.182, *p* = .671, ηp^2^ = .001) | UK | 4.88 |  | ***UK****: -*0.137 (*F* = 0.372, *p* = .543, ηp^2^ = .002)  ***Non-UK:*** 0.274 (*F* = 1.221, *p* = .271, ηp^2^ = .006) |  |  |  |  |  |  |
|  |  |  |  | Non-UK | 5.13 |  |  |  |  |  |  |  |  |
|  | Time-2 | 5.08 |  | UK | 4.75 |  |  |  |  |  |  |  |  |
|  |  |  |  | Non-UK | 5.40 |  |  |  |  |  |  |  |  |
| Reflection – Intention | | | | | | | | | | | | | |
| Control | Time-1 | 6.02 | **-0.313 (*F* = 4.122, *p* = .044, ηp^2^ = .020)** | UK | 5.80 | ***UK T-1****:* 0.134 (*F* = 0.457, *p* = .500, ηp^2^ = .002)  ***UK T-2:* 0.447 (*F* = 5.298, *p* = .022, ηp^2^ = .026)**  ***Non-UK T-1****:*  0.029 (*F* = 0.007, *p* = .932, ηp^2^ < .001)  ***Non-UK T-2:*** **0.707 (*F* = 4.453, *p* = .036, ηp^2^ = .022)** | ***UK****: -*0.141 (*F* = 1.230, *p* = .269, ηp^2^ = .006)  ***Non-UK:*** -0.486 (*F* = 3.038, *p* = .083, ηp^2^ = .015)  ***UK****:* 0.172 (*F* = 1.372, *p* = .243, ηp^2^ = .007)  ***Non-UK:*** 0.192 (*F* = 1.387, *p* = .240, ηp^2^ = .007) | F(1,198) = 0.288, *p* = .592, ηp^2^ = .001 | *F*(1,198) = 2.851, *p* = .093, ηp^2^ = .014 | *F*(1,198) = 3.519, *p* = .062, ηp^2^ = .017 | ***F*(1,198) = 6.557, *p* = .011, ηp^2^ = .032** | *F*(1,198) = 0.980, *p* = .323, ηp^2^ = .005 |  |
|  |  |  |  | Non-UK | 6.24 |  |  |  |  |  |  |  |  |
|  | Time-2 | 5.71 |  | UK | 5.66 |  |  |  |  |  |  |  |  |
|  |  |  |  | Non-UK | 5.75 |  |  |  |  |  |  |  |  |
| Intervention | Time-1 | 6.10 | 0.182 (*F* = 2.940, *p* = .088, ηp^2^ = .015) | UK | 5.94 |  |  |  |  |  |  |  |  |
|  |  |  |  | Non-UK | 6.27 |  |  |  |  |  |  |  |  |
|  | Time-2 | 6.28 |  | UK | 6.11 |  |  |  |  |  |  |  |  |
|  |  |  |  | Non-UK | 6.46 |  |  |  |  |  |  |  |  |
| Confidentiality – Attitude | | | | | | | | | | | | | |
| Control | Time-1 | 4.59 | 0.053 (*F* = 0.146, *p* = .703, ηp^2^ = .001) | UK | 4.76 | ***UK T-1****:* -0.328 (*F* = 2.683, *p* = .103, ηp^2^ = .013)  ***UK T-2:* 0.376 (*F* = 4.052, *p* = .045, ηp^2^ = .020)**  ***Non-UK T-1****:*  0.201 (*F* = 0.338, *p* = .562, ηp^2^ = .002)  ***Non-UK T-2:*** 0.324 (*F* = 1.003, *p* = .318, ηp^2^ = .005) | ***UK****: -*0.173 (*F* = 2.286, *p* = .132, ηp^2^ = .011)  ***Non-UK:*** 0.279 (*F* = 1.241, *p* = .267, ηp^2^ = .006) | F(1,197) = 1.897, *p* = .170, ηp^2^ = .010 | *F*(1,197) = 0.003, *p* = .958, ηp^2^ < .001 | *F*(1,197) = 0.647, *p* = .422, ηp^2^ = .003 | ***F*(1,197) = 5.596, *p* = .019, ηp^2^ = .028** | *F*(1,197) = 3.067, *p* = .081, ηp^2^ = .015 |  |
|  |  |  |  | Non-UK | 4.42 |  |  |  |  |  |  |  |  |
|  | Time-2 | 4.65 |  | UK | 4.59 |  |  |  |  |  |  |  |  |
|  |  |  |  | Non-UK | 4.70 |  |  |  |  |  |  |  |  |
| Intervention | Time-1 | 4.53 | **0.466 (*F* = 23.513, *p* < .001, ηp^2^ = .107)** | UK | 4.43 |  | ***UK****:* **0.531 (*F* = 16.176, *p* < .001, ηp^2^ = .076)**  ***Non-UK:* 0.401 (*F* = 7.272, *p* = .036, ηp^2^ = .036)** |  |  |  |  |  |  |
|  |  |  |  | Non-UK | 4.63 |  |  |  |  |  |  |  |  |
|  | Time-2 | 4.99 |  | UK | 4.96 |  |  |  |  |  |  |  |  |
|  |  |  |  | Non-UK | 5.03 |  |  |  |  |  |  |  |  |
| Confidentiality – Subjective norms | | | | | | | | | | | | | |
| Control | Time-1 | 4.28 | -0.247 (*F* = 1.476, *p* = .226, ηp^2^ = .007) | UK | 3.98 | ***UK T-1****:* 0.207 (*F* = 0.603, *p* = .439, ηp^2^ = .003)  ***UK T-2:*** 0.293 (*F* = 1.130, *p* = .289, ηp^2^ = .006)  ***Non-UK T-1****:*  -0.157 (*F* = 0.115, *p* = .735, ηp^2^ = .001)  ***Non-UK T-2:*** **1.013 (*F* = 4.511, *p* = .035, ηp^2^ = .022)** | ***UK****:* 0.121 (*F* = 0.521, *p* = .471, ηp^2^ = .003)  ***Non-UK:*** -0.616 (*F* = 2.802, *p* = .096, ηp^2^ = .014) | F(1,196) = 0.667, *p* = .415, ηp^2^ = .003 | *F*(1,196) = 1.533, *p* = .217, ηp^2^ = .008 | *F*(1,196) = 1.894, *p* = .170, ηp^2^ = .010 | ***F*(1,196) = 5.599, *p* = .015, ηp^2^ = .030** | ***F*(1,196) = 4.937, *p* = .027, ηp^2^ = .025** |  |
|  |  |  |  | Non-UK | 4.57 |  |  |  |  |  |  |  |  |
|  | Time-2 | 4.03 |  | UK | 4.10 |  |  |  |  |  |  |  |  |
|  |  |  |  | Non-UK | 3.95 |  |  |  |  |  |  |  |  |
| Intervention | Time-1 | 4.30 | **0.381 (*F* = 7.210, *p* = .008, ηp^2^ = .035)** | UK | 4.19 |  | ***UK****:* 0.207 (*F* = 1.138, *p* = .287, ηp^2^ = .006)  ***Non-UK:*** **0.555 (*F* = 6.378, *p* = .012, ηp^2^ = .032)** |  |  |  |  |  |  |
|  |  |  |  | Non-UK | 4.41 |  |  |  |  |  |  |  |  |
|  | Time-2 | 4.68 |  | UK | 4.40 |  |  |  |  |  |  |  |  |
|  |  |  |  | Non-UK | 4.97 |  |  |  |  |  |  |  |  |
| Confidentiality – Perceived behaviour control | | | | | | | | | | | | | |
| Control | Time-1 | 4.40 | -0.228 (*F* = 2.670, *p* = .104, ηp^2^ = .013) | UK | 4.51 | ***UK T-1****:* -0.135 (*F* = 0.425, *p* = .515, ηp^2^ = .002)  ***UK T-2:* 0.531 (*F* = 7.397, *p* = .007, ηp^2^ = .036)**  ***Non-UK T-1****:*  0.328 (*F* = 0.837, *p* = .361, ηp^2^ = .004)  ***Non-UK T-2:*** **1.208 (*F* = 12.784, *p* < .001, ηp^2^ = .061)** | ***UK****: -*0.213 (*F* = 3.464, *p* = .064, ηp^2^ = .017)  ***Non-UK:*** -0.242 (*F* = 0.924, *p* = .338, ηp^2^ = .005) | F(1,196) = 0.003, *p* = .958, ηp^2^ < .001 | *F*(1,196) = 0.095, *p* = .758, ηp^2^ < .001 | ***F*(1,196) = 6.705, *p* = .010, ηp^2^ = .033** | ***F*(1,196) = 19.396, *p* < .001, ηp^2^ = .090** | *F*(1,196) = 0.412, *p* = .522, ηp^2^ = .002 |  |
|  |  |  |  | Non-UK | 4.29 |  |  |  |  |  |  |  |  |
|  | Time-2 | 4.17 |  | UK | 4.29 |  |  |  |  |  |  |  |  |
|  |  |  |  | Non-UK | 4.05 |  |  |  |  |  |  |  |  |
| Intervention | Time-1 | 4.50 | **0.545 (*F* = 31.569, *p* < .001, ηp^2^ = .139)** | UK | 4.37 |  | ***UK****:* **0.452 (*F* = 11.635, *p* = .001, ηp^2^ = .056)**  ***Non-UK:* 0.638 (*F* = 18.018, *p* < .001, ηp^2^ = .084)** |  |  |  |  |  |  |
|  |  |  |  | Non-UK | 4.62 |  |  |  |  |  |  |  |  |
|  | Time-2 | 5.04 |  | UK | 4.82 |  |  |  |  |  |  |  |  |
|  |  |  |  | Non-UK | 5.26 |  |  |  |  |  |  |  |  |
| Confidentiality – Intentions | | | | | | | | | | | | | |
| Control | Time-1 | 5.10 | **-0.362 (*F* = 4.009, *p* = .047, ηp^2^ = .020)** | UK | 4.88 | ***UK T-1****:* 0.215 (*F* = 0.902, *p* = .343, ηp^2^ = .005)  ***UK T-2:* 0.888 (*F* = 15.385, *p* < .001, ηp^2^ = .072)**  ***Non-UK T-1****:*  0.511 (*F* = 1.691, *p* = .195, ηp^2^ = .009)  ***Non-UK T-2:* 1.111 (*F* = 8.031, *p* = .005, ηp^2^ = .039)** | ***UK****:* ***-*0.338 (*F* = 5.134, *p* = .025, ηp^2^ = .025)**  ***Non-UK:*** -0.387 (*F* = 1.403, *p* = .238, ηp^2^ = .007) | F(1,197) = 0.041, *p* = .840, ηp^2^ < .001 | ***F*(1,197) = 7.349, *p* = .007, ηp^2^ = .036** | ***F*(1,197) = 11.336, *p* = .001, ηp^2^ = .054** | ***F*(1,197) = 7.827, *p* = .006, ηp^2^ = .038** | *F*(1,197) = 0.028, *p* = .867, ηp^2^ < .001 |  |
|  |  |  |  | Non-UK | 5.32 |  |  |  |  |  |  |  |  |
|  | Time-2 | 4.74 |  | UK | 4.54 |  |  |  |  |  |  |  |  |
|  |  |  |  | Non-UK | 4.93 |  |  |  |  |  |  |  |  |
| Intervention | Time-1 | 5.46 | **0.274 (*F* = 4.797, *p* = .030, ηp^2^ = .024)** | UK | 5.09 |  | ***UK****:* 0.335 (*F* = 3.793, *p* = .053, ηp^2^ = .019)  ***Non-UK:*** 0.214 (*F* = 1.214, *p* = .272, ηp^2^ = .006) |  |  |  |  |  |  |
|  |  |  |  | Non-UK | 5.83 |  |  |  |  |  |  |  |  |
|  | Time-2 | 5.74 |  | UK | 5.43 |  |  |  |  |  |  |  |  |
|  |  |  |  | Non-UK | 6.05 |  |  |  |  |  |  |  |  |

*Table 2*. Changes in the theory of planned behaviour factors of three professional behaviours after the intervention and three months after

| **Behaviour** | **Scale/measure** | **Time point** | **Mean scores** | **Time effect** |
| --- | --- | --- | --- | --- |
| Use of confidentiality guidance | Attitudes | Time-1 | **4.42^a,c^** | ***F*(2,74) = 6.372, *p* = .003, ηp^2^ = .147** |
|  |  | Time-2 | **4.97^a^** |  |
|  |  | Time-3 | **4.85^c^** |  |
|  | Subjective norms | Time-1 | 4.02 | *F*(2,74) = 2.154, *p* = .123, ηp^2^ = .055 |
|  |  | Time-2 | 4.42 |  |
|  |  | Time-3 | 4.37 |  |
|  | Perceived behaviour control | Time-1 | **4.29^a^** | ***F*(2,74) = 8.827, *p* < .001, ηp^2^ = .193** |
|  |  | Time-2 | **4.92^a^** |  |
|  |  | Time-3 | 4.68 |  |
|  | Intentions | Time-1 | 5.15 | *F*(2,74) = 2.042, *p* = .137, ηp^2^ = .052 |
|  |  | Time-2 | 5.45 |  |
|  |  | Time-3 | 5.45 |  |
| Raising concerns | Attitudes | Time-1 | **4.13^a^** | ***F*(2,74) = 5.022, *p* = .009, ηp^2^ = .120** |
|  |  | Time-2 | **4.64^a,b^** |  |
|  |  | Time-3 | **4.20^b^** |  |
|  | Subjective norms | Time-1 | 4.78 | *F*(2,74) = 0.142, *p* = .868, ηp^2^ = .004 |
|  |  | Time-2 | 4.76 |  |
|  |  | Time-3 | 4.84 |  |
|  | Perceived behaviour control | Time-1 | 5.20 | *F*(2,74) = 0.530, *p* = .591, ηp^2^ = .014 |
|  |  | Time-2 | 5.30 |  |
|  |  | Time-3 | 5.11 |  |
|  | Intentions | Time-1 | 5.50 | *F*(2,74) = 0.179, *p* = .836, ηp^2^ = .005 |
|  |  | Time-2 | 5.58 |  |
|  |  | Time-3 | 5.46 |  |
| Reflective practice | Attitudes | Time-1 | 5.24 | *F*(2,74) = 0.542, *p* = .584, ηp^2^ = .014 |
|  |  | Time-2 | 5.38 |  |
|  |  | Time-3 | 5.27 |  |
|  | Subjective norms | Time-1 | **4.50^a,c^** | ***F*(2,74) = 6.337, *p* = .003, ηp^2^ = .146** |
|  |  | Time-2 | **4.91^a^** |  |
|  |  | Time-3 | **4.88^c^** |  |
|  | Perceived behaviour control | Time-1 | 4.95 | *F*(2,74) = 1.375, *p* = .259, ηp^2^ = .036 |
|  |  | Time-2 | 5.21 |  |
|  |  | Time-3 | 4.79 |  |
|  | Intentions | Time-1 | 5.96 | *F*(2,74) = 2.882, *p* = .062, ηp^2^ = .072 |
|  |  | Time-2 | 6.29 |  |
|  |  | Time-3 | 5.94 |  |

*Note*. ^a^ significant changes between Time-1 and Time-2 measure, ^b^ significant changes between Time-2 and Time-3 measure, ^c^ significant changes between Time-1 and Time-3 measure.
