# Supplementary material for: Changing professional behaviours: mixed methods study utilising psychological theories to evaluate an educational programme for UK medical doctors
Source: BMC Med Educ. 2021 Feb 5;21:92. doi: 10.1186/s12909-021-02510-4 (PMC7866444; doi:10.1186/s12909-021-02510-4)
Supplement: Supplementary file 6 — Additional file 6. [file 12909_2021_2510_MOESM6_ESM.docx]

# Supplementary File 5

*Table 1*. Additional analysis

| **Measure** | | **Additional analysis** |
| --- | --- | --- |
| Use of confidentiality guidance | Intention | There was no significant difference in this measure between intervention and control groups at Time-1 (*F* = 2.442, *p* = .120, ηp^2^ = .012) but there was a significant difference at Time-2 (*F* = 18.573, *p* < .001, ηp^2^ = .086, part of split-plot ANCOVA analysis). We also performed linear regression predicting the change score from Time-2 to Time-1, controlling for primary medical qualification (PMQ) and years of experience. This analysis showed that group (intervention/control) was a significant predictor of the change score (*p* < .05). |
| Raising concerns | Intention | There was no significant difference in intentions to raise concerns between intervention and control groups at Time-1 (*F* = 1.017, *p* = .314, ηp^2^ = .005) or at Time-2 (*F* = 0.642, *p* = .424, ηp^2^ = .003; part of split-plot ANCOVA analysis). The regression analysis predicting the change score between Time-2 and Time-1 (controlling for PMQ and years of experience) revealed that group (intervention/control) was not a significant predictor of intentions to raise concerns (*p* > .05). |
| Reflection | Attitudes | There was no significant difference in attitudes towards reflection between intervention and control groups at Time-1 (*F* < 0.001, *p* = .994, ηp^2^ < .001) or Time-2 (*F* = 3.392, *p* = .067, ηp^2^ = .017; part of split-plot ANCOVA analysis). Group (intervention/control) significantly predicted (p < .05, linear regression) the change score between Time-2 and Time-1 (controlling for PMQ and years of experience). |
| Reflection | Intention | There was no significant difference in intentions to reflect between intervention and control groups at Time-1 (*F* = 0.163, *p* = .687, ηp^2^ = .001) but there was a significant difference at Time-2 (*F* = 8.471, *p* = .004, ηp^2^ = .041; part of split-plot ANCOVA analysis). The change score between Time-2 and Time-1 was non-normally distributed and, therefore, the non-parametric Mann-Witney test was used to test the difference between two groups. It showed that there was no significant difference between intervention and control groups in the change scores (*p* > .05). However, this analysis does not include control variables (PMQ and years of experience). Therefore, we removed five outliers making the change scores normally distributed and performed linear regression. This analysis showed that group (intervention/control) predicted the change over time in intentions towards reflection (*p* < .05). |
